# COVID-19 Collateral Damage: Management of Periprosthetic Joint Infection in Malaysia

**DOI:** 10.7759/cureus.18820

**Published:** 2021-10-16

**Authors:** Mohammed Harris Anwarali Khan, Ren Yi Kow, Sasidaran Ramalingam, Jade Pei Yuik Ho, Jeffrey Jaya Raj, Kunalan Ganthel@Annamalai, Chooi Leng Low

**Affiliations:** 1 Department of Orthopaedics, Hospital Kuala Lumpur, Kuala Lumpur, MYS; 2 Department of Orthopaedics, Traumatology & Rehabilitation, International Islamic University Malaysia, Kuantan, MYS; 3 Department of Orthopaedics, Hospital Tengku Ampuan Afzan, Kuantan, MYS; 4 Department of Radiology, International Islamic University Malaysia, Kuantan, MYS

**Keywords:** periprosthetic joint infection, covid 19, implant failure, infection, revision surgery

## Abstract

Background and objective

Periprosthetic joint infection (PJI) is one of the dreaded complications in patients after arthroplasty surgeries, owing to the risk of morbidity and arduous investigations and management associated with it. Nevertheless, as Malaysia is currently battling against the severe acute respiratory syndrome coronavirus 2 (SARS-CoV-2)-induced coronavirus disease 2019 (COVID-19) pandemic head-on, the treatment for other non-life-threatening diseases including PJI has taken a backseat. In this study, we present a case series of 11 patients with PJI who were managed surgically at the largest tertiary hospital in Malaysia and we hope to shed some light on the difficulties we have encountered during this trying period.

Patients and methods

Patients with PJIs who underwent surgical intervention during the ongoing COVID-19 pandemic (March 1, 2020, to June 30, 2021) were reviewed and included in this study. The demographic profile of the patients, presenting complaints, prosthesis topography, biochemical investigative findings, surgical interventions, and short-term outcomes were summarized.

Results

A total of 11 patients were treated surgically at Hospital Kuala Lumpur for PJI. Among them, five patients are still awaiting their second-stage surgeries despite the completion of their antibiotic regimes, and they are fit for the procedure.

Conclusion

The COVID-19 pandemic has wreaked havoc on the treatment of patients with PJI. In a setting with scarce resources, surgeons should strongly consider single-stage revision surgeries for the treatment of patients with PJI.

## Introduction

As the severe acute respiratory syndrome coronavirus 2 (SARS-CoV-2)-induced coronavirus disease 2019 (COVID-19) pandemic sweeps through the whole world like a wildfire, crumbling most of the healthcare and other crucial sectors along the way, many countries are scrambling to manage and contain this debilitating disease [1-3]. As the healthcare system is stretched to the brink of collapse in many countries, the management of COVID-19 patients has taken precedence over other patients [1-3]. Elective surgical interventions have been postponed indefinitely, ranging from those for patients with cardiac diseases to patients with surgical illnesses [1-3]. While the administrators are justified in focusing on COVID-19 infections and associated mortality rates, the collateral damage of COVID-19 infection in the healthcare sector remains obscured to the public.

In Malaysia, elective surgeries such as joint replacement surgeries have been postponed throughout this pandemic period in many tertiary centers. Nevertheless, complications of joint replacement surgeries, such as periprosthetic joint infection (PJI), are considered to be orthopaedic emergencies, which require urgent surgical intervention. In patients who undergo primary joint replacement, the infection rate in the first two years usually ranges from 0.5 to 2% and revision surgeries normally have higher rates of infection, ranging from 2.5 to 4% [4,5]. PJI is a devastating condition associated with a high rate of mortality, increased risk of morbidity, decreased quality of life, and potentially decreased level of mobility and ambulation [4,5]. Common risk factors for joint replacement surgery are postoperative surgical site infection, previous arthroplasty, advanced age, malnutrition, joint disease, obesity, diabetes mellitus, and remote infection [4-6]. Management of PJI can be done via single- or two-stage revision surgery, with each having its pros and cons [7,8]. During the COVID-19 pandemic, surgeons have to improvise and adjust accordingly, depending on the surge of patients infected with the SARS-CoV-2 virus. We present a case series of 11 patients with PJI who were managed surgically during the ongoing pandemic at the largest tertiary hospital in Malaysia and we hope to highlight the difficulties we have encountered during this trying period.

## Materials and methods

All patients who were treated surgically at Hospital Kuala Lumpur from March 1, 2020, to June 30, 2021, for PJI were included in this study. Written consents were obtained from all patients for the publication of this article. They were diagnosed to have PJI based on the Musculoskeletal Infection Society (MSIS) criteria, whereby at least one of the following criteria was present (Table 1) [8].

**Table 1:** Periprosthetic joint infection diagnosis based on the Musculoskeletal Infection Society (MSIS) criteria

Periprosthetic joint infection diagnosis based on the Musculoskeletal Infection Society (MSIS) criteria, whereby at least one of the following criteria was present		
1	Presence of sinus tract communicating with the prosthesis	
2	A microorganism was isolated from the affected joint from at least two separate tissues or synovial fluid samples	
3	Four out of six following criteria were fulfilled	
	A	Raised serum erythrocyte sedimentation rate (ESR) and serum C-reactive protein (CRP)
	B	Elevated synovial white blood cell count
	C	Raised synovial neutrophil percentage
	D	Presence of pus in the affected joint
	E	Isolation of a pathogen in one culture of periprosthetic tissue or synovial fluid
	F	More than five neutrophils per high-power field from histologic analysis of affected joint tissue

All patients were followed up for a minimum of four months after the surgical intervention. The demographic data, investigative results, and types of surgical interventions were obtained from the medical records. Patients who did not fulfill the MSIS criteria or those who were lost to follow-up were excluded from this case series.

## Results

A total of 11 patients were enrolled in this study, with ages ranging from 39 to 79 years. Most patients were males (n=7, 63.6%) with Chinese being the most predominant ethnicity (n=6, 54.5%). All patients had pre-existing comorbidities, with a majority of them having identifiable risk factors (n=9, 81.8%). Most of the PJIs involved the knee joint (n=7, 63.6%), followed by the hip joint (three total hip replacements and one bipolar hemiarthroplasty). Their presenting complaint was mostly pain or pain with joint swelling with a variable duration ranging from two days to six months.

Table 2 summarizes the patients' demographics (age, gender, body mass index, smoking status, comorbidities, and other risk factors), their presenting complaints and duration, and the affected joint topography.

**Table 2:** Patients' demographic data, risk factors, presenting complaints and duration, and the affected joint topography M: male; F: female; BMI: body mass index; DM: diabetes mellitus; THR: total hip replacement; TKR: total knee replacement

Patient	Age (years)	Gender	Ethnicity	BMI (kg/m^2^)	Smoking status	Comorbidities	Risk factors	Signs and symptoms	Duration of symptoms	Duration from index surgery	Prosthesis topography	Initial indication for replacement surgery
1	74	F	Chinese	30	No	DM, hyperlipidemia, rheumatoid arthritis	Steroid treatment	Right hip pain	6 months	14 years	Right THR	Osteoarthritis
2	39	M	Indian	26.6	No	Rheumatoid arthritis, Cushing's syndrome, Hepatitis B	Steroid treatment, hepatitis B	Right knee pain with sinus discharge	1 week	6 years	Right TKR	Osteoarthritis
3	70	F	Chinese	25	No	Hypertension	History of cellulitis	Right knee pain and swelling	1 month	2 years	Right TKR	Osteoarthritis
4	75	M	Chinese	27.7	No	Hypertension	Nil	Left knee pain	1 month	2 years	Left TKR	Osteoarthritis
5	68	F	Indian	24.2	No	Hypertension, chronic renal disease	Chronic renal disease	Right knee swelling	1 month	4 years	Right TKR	Osteoarthritis
6	79	F	Chinese	30	No	DM, hypertension, hyperlipidemia	DM	Right knee pain	2 days	1 year	Right TKR	Osteoarthritis
7	63	M	Malay	30	No	Hypertension, chronic renal disease	Chronic renal disease	Left hip pain	6 months	10 years	Left THR	Osteoarthritis
8	66	F	Malay	32	No	Hypertension	History of cellulitis	Right knee pain	2 weeks	10 years	Right TKR	Osteoarthritis
9	74	F	Chinese	16.6	No	Hypertension, hyperlipidemia	Delayed suture removal	Left hip pain and swelling	10 days	2 months	Left bipolar hemiarthroplasty	Neck of femur fracture
10	56	M	Chinese	28.4	No	Hypertension	Nil	Right hip pain and swelling	3 months	1 year	Right THR	Osteoarthritis
11	53	F	Malay	30	No	DM, hypertension	Skin infection	Right knee pain and swelling	3 months	1 year	Right TKR	Osteoarthritis

Table 3 illustrates the patients’ investigation results such as serum parameters (white cell count, C-reactive protein level, and erythrocyte sedimentation rate), plain radiographic findings, samples obtained and pathogens isolated, and their subsequent surgical intervention and chemotherapy details.

**Table 3:** Patients' investigation results, surgical intervention and antibiotic therapy, and their short-term outcomes ESBL: extended-spectrum beta-lactamase; CONS: coagulase-negative staphylococci; WD: wound debridment; IR: implant removal; JW: joint washout; IV: intravenous; Augmentin: amoxicillin/clavulanate; Bactrim: trimethoprim/sulfamethoxazole; Unasyn: ampicillin/sulbactam

Patient	WBC (10^9^/L)	CRP (mg/L)	ESR (mm/hr)	X-ray findings	Sample(s)	Microorganism isolated	First-stage surgery	Second-stage surgery	Antibiotic usage	Short-term outcome	Comment
1	7.9	3.7	47	Implant loosening at the acetabulum and femoral component with osteomyelitis at the proximal femur	Synovial fluid, tissue, and bone	No growth	WD, IR, and cement spacer with interlocking nail	Pending	IV cloxacillin for 6 weeks then oral cloxacillin for 3 months	The wound healed, wheelchair ambulating	
2	10.7	6.7	43	No implant loosening	Tissue and pus	ESBL	WD, JW (two times)	NA	IV meropenem for 6 weeks	The wound healed, ambulating with a walking aid	The patient refuses the removal of the implant
3	4.1	5.5	39	No implant loosening	Synovial fluid	No growth	WD, JW, and insert exchange	NA	IV ciprofloxacin and clindamycin for 1 week then oral Bactrim and rifampicin for 3 months	The wound healed, ambulating without walking aid	
4	9.3	56.6	115	No implant loosening	Synovial fluid	CONS	WD, JW, and cement spacer	Pending	IV vancomycin for 2 weeks then oral Bactrim and clindamycin for 3 months	The wound healed, ambulating without walking aid	
5	7.7	105	117	No implant loosening	Synovial fluid, tissue, and bone	Acinetobacter baumanii	WD, JW, and cement spacer	Pending	IV Augmentin for 2 weeks then oral Unasyn for 3 months	The wound healed, partially ambulating with a walking aid	
6	8	3.1	63	No implant loosening	Synovial fluid	Staphylococcus aureus	WD, JW, and cement spacer	NA	IV cloxacillin for 2 weeks then oral cloxacillin and fusidic acid for 6 weeks	The wound healed, ambulating without walking aid	
7	9	3.4	26	No implant loosening	Synovial fluid	CONS	WD, JW, and insert exchange	Pending	IV clindamycin for 2 weeks then oral Unasyn for 3 months	The wound healed, ambulating with non-weight-bearing clutches	
8	10	161	120	Implant loosening at the femoral and tibial component	Synovial fluid	Streptococcus dysgalactiae	WD, JW, and insert exchange	NA	IV penicillin for 2 weeks then oral Unasyn for 10 weeks	The wound healed, ambulating with a walking aid	
9	8.86	20	66	No implant loosening	Synovial fluid	Gram-positive bacilli	WD, JW, and Collatamp insertion	NA	Oral Bactrim and oral doxycycline for 6 months	The wound healed, ambulating with a walking aid	
10	7.7	84	106	No implant loosening	Tissue	Serratia sp.	WD, JW, and exchange of femoral head component	NA	IV cefepime for 6 weeks then oral ciprofloxacin for 6 weeks	The wound healed, ambulating with a walking aid	
11	10	35	117	No implant loosening	Synovial fluid	Staphylococcus aureus	WD, IR, JW, and cement spacer	Pending	IV cloxacillin for 2 weeks then oral cloxacillin for 3 months	The wound healed, partially ambulating with a walking aid	

Most of the patients’ serum white blood cell counts were not elevated, ranging from 4.1 to 10.7 x 10^9^/L. Similarly, only five patients (45.4%) had raised serum C-reactive protein. Nevertheless, all patients except one had elevated erythrocyte sedimentation rates. Upon reviewing the plain radiographs, only two patients had signs to suggest implant loosening affecting the hip and knee joints respectively. In terms of sample collection, no antibiotic was given two weeks prior to the collection of intraoperative cultures as suggested by the American Academy of Orthopaedic Surgeons (AAOS) [9]. Tissue and bone samples were directly sent to the laboratory while synovial fluid samples were sent in blood culture bottles (BACTEC^TM^,^ ^Becton Dickinson Diagnostic Instrument Systems, Sparks, MD). All except two samples yielded positive cultures with various pathogens isolated.

Five patients (45.4%) underwent wound debridement, implant removal, and antibiotic cement spacer insertion, while another five patients (45.4%) underwent single-stage surgeries. One patient refused implant removal and underwent wound debridement and joint washout twice. Despite the completion of antibiotics with subsequent improvement in septic parameters, their second-stage surgeries were postponed indefinitely due to the COVID-19 pandemic affecting the availability of operation theatres. Among the patients who underwent single-stage surgeries, two were able to ambulate without a walking aid while another three patients were ambulating with a walking aid.

## Discussion

PJI remains a significant challenge for arthroplasty surgeons in terms of diagnosis as well as management [9]. A combination of detailed clinical findings, blood and radiological investigations, coupled with synovial or tissue culture are required to properly diagnose a PJI. In the midst of the ongoing COVID-19 pandemic, patients with SARS-CoV-2 infection can present with atypical symptoms masquerading as PJI to further complicate the process of PJI diagnosis [10]. The dilemma we encounter was represented by case 8 in this series, wherein the patient who had undergone a bilateral total knee replacement 11 years ago presented with fever for one day associated with right knee pain and swelling for two weeks. She had a history of right lower limb cellulitis requiring admission and intravenous antibiotics two years ago. Clinically, her right knee was warm and tender with signs of effusion and limited range of movement. Her septic parameters were all elevated, further suggesting a PJI. Nevertheless, she had been in close contact with a patient with the COVID-19 infection, making her a person-under-investigation (PUI). While waiting for her polymerase chain reaction (PCR) result, arthrocentesis from the affected joint yielded Gram-positive pathogens. As she was relatively stable, she was scheduled for surgery after the period of quarantine and a repeat PCR test. She subsequently underwent wound debridement, synovectomy, joint washout, and polyethylene insert exchange.

Besides the admitted patients being at risk of COVID-19 infection, as the COVID-19 pandemic stretches the healthcare resources to the brink of collapse, most of the hospitals place their focus solely on treating patients with life-threatening conditions. All elective surgeries have been postponed to channel the resources, in terms of manpower, bed availability, and medications, toward the management of patients with SARS-CoV-2 infection. This has created an imbroglio, wherein patients who have undergone first-stage surgery and have completed antibiotic chemotherapy have to wait indefinitely for the second-stage surgery as COVID-19 shows no sign of abating. Throughout this pandemic period, there have been five patients in our center who are still awaiting their second-stage surgeries despite the completion of their antibiotic regimens and although their septic parameters are appropriate for second-stage surgery. Patients who have had first-stage revision are initially planned for revision surgeries six weeks after their first-stage surgeries. In view of the delay in the second-stage surgery, we have decided to stop antibiotics three months after the first-stage surgery despite the fact that the routine practice in our center is to continue antibiotics until the second-stage surgery. A comparative analysis of different issues encountered during the COVID-19 pandemic versus the pre-pandemic period regarding the management of patients with PJI is presented in Table 4.

**Table 4:** Comparison of different issues encountered during the COVID-19 pandemic and pre-pandemic period for the management of patients with PJI PJI: periprosthetic joint infection; COVID-19: coronavirus disease 2019; PUI: person under investigation

Issues encountered	Pre-pandemic period	During the pandemic
Patients’ presentation	Early (usually within days to weeks upon symptoms occurrence)	Delayed (mean of 60 days in this series)
Investigations	Routine septic investigations (biochemical, culture, and radiographic investigations)	Routine septic investigations PLUS COVID-19 polymerase chain reaction (PCR)
Bed management	Generally separated from patients who have undergone clean surgeries	Generally separated from patients with PUI status. Separation from patients with clean surgeries cannot be guaranteed
Management (first-stage surgery)	Options of wound debridement, joint washout, and insert exchange or implant removal and cement spacer insertion, depending on intraoperative findings	Single-stage surgery such as wound debridement, joint washout, and insert exchange is preferred if clinically indicated
Management (antibiotic regimen)	Generally given for 6 to 8 weeks until second-stage surgery (based on culture and sensitivity)	Given for a maximum of 3 months (while waiting for second-stage surgery)
Management (second-stage surgery)	Performed when clinically indicated (favorable soft tissue conditions and improved septic parameters)	Postponed indefinitely due to the COVID-19 pandemic

In this difficult time, the question remains as to how to optimize the recovery of patients with PJI without increasing the burden on the healthcare system. This prompts us to favor single-stage revision surgery instead of the cumbersome two-stage revision surgery. There is now evidence from several comparative case series that complications and reinfection rates after single-stage revisions are similar to those in two-stage revisions [11-14]. Revision surgery protocols of PJI, designed to address the nature of these infections, were first introduced over three decades ago. The fundamental concepts for the treatment of PJI entail the necessity for implants, foreign bodies, and cement removal in combination with radical debridement. This is coupled with the use of antibiotic-loaded cement. It is based on these principles that today’s limb salvage protocols have evolved. Surgeons are now able to take advantage of growing technological innovations and employ aggressive surgical techniques for debridement followed by the restoration of function in patients with PJI. The surge in COVID-19 cases has shortened the operation list and surgery time is significantly reduced. Wise decisions should be made and resources available should be utilized smartly and judiciously to provide the best care to patients. In our center, those who underwent single-stage surgeries have achieved relatively good short-term outcomes.

Limitations of the study

This study was limited by the small number of patients included from a single institution. Nevertheless, this was the first-of-its-kind study to shed some light on an important aspect of the COVID-19 collateral damage.

## Conclusions

The COVID-19 pandemic has caused unmeasurable collateral damage to the healthcare system globally. Many patients suffering from non-COVID-19-related diseases have received sub-standard treatment or have had their treatment delayed. It is paramount for healthcare workers to be equipped with the relevant knowledge and clinical wisdom in treating patients during this COVID-19 pandemic. Surgeons should always maintain the highest clinical awareness and level of suspicion in terms of recognizing signs and symptoms of PJI. In the midst of the COVID-19 pandemic, surgeons should strongly consider the option of single-stage surgery for patients with PJI whenever feasible.
